# Regulation of microglia phagocytosis and potential involvement of exercise

**DOI:** 10.3389/fncel.2022.953534

**Published:** 2022-07-25

**Authors:** Congqin Li, Yong Wang, Ying Xing, Jing Han, Yuqian Zhang, Anjing Zhang, Jian Hu, Yan Hua, Yulong Bai

**Affiliations:** ^1^Department of Rehabilitation Medicine, Huashan Hospital, Fudan University, Shanghai, China; ^2^Department of Neurology, Minhang Hospital, Fudan University, Shanghai, China; ^3^State Key Laboratory of Medical Neurobiology, Department of Integrative Medicine and Neurobiology, Brain Science Collaborative Innovation Center, School of Basic Medical Sciences, Institutes of Brain Science, Fudan Institutes of Integrative Medicine, Fudan University, Shanghai, China; ^4^National Center for Neurological Disorders, Huashan Hospital, Fudan University, Shanghai, China; ^5^National Clinical Research Center for Aging and Medicine, Huashan Hospital, Fudan University, Shanghai, China

**Keywords:** microglia, phagocytosis, exercise, TREM2, synapsis

## Abstract

Microglia are considered the main phagocytic cells in the central nervous system, remodeling neural circuits by pruning synapses during development. Microglial phagocytosis is also a crucial process in maintaining adult brain homeostasis and clearing potential toxic factors, which are recognized to be associated with neurodegenerative and neuroinflammatory disorders. For example, microglia can engulf amyloid-β plaques, myelin debris, apoptotic cells, and extracellular harmful substances by expressing a variety of specific receptors on the cell surface or by reprogramming intracellular glucose and lipid metabolism processes. Furthermore, physical exercise has been implicated to be one of the non-pharmaceutical treatments for various nervous system diseases, which is closely related to neuroplasticity and microglia functions including proliferation, activation, and phagocytosis. This review focuses on the central regulatory mechanisms related to microglia phagocytosis and the potential role of exercise training in this process.

## Introduction

Microglia are the innate immune cells of the central nervous system (CNS) and have a long life span, accounting for 5–20% of glial cells in the adult brain (Mecca et al., 2018). They are malleable in response to different signals (Wendeln et al., 2018) and can undergo extensive morphological, phenotypic, and functional reprogramming to adapt to the changing needs of the developing brain. Microglia play a variety of roles in the brain such as immune monitoring, secretion of cytokines and neurotrophic factors, regulation of inflammation, phagocytosis of cell debris, synaptic connection and pruning, and neural circuit remodeling under physiological and pathological conditions (Kettenmann et al., 2011; Hayashi and Nakanishi, 2013). In addition, microglia are considered crucial regulators of CNS development and homeostasis through the crosstalk with other glia and neurons (Colonna and Butovsky, 2017; Hagemeyer et al., 2017).

In a physiological state, microglia show a highly branched state with a small cell body and long branches, which is called “resting microglia.” They continuously extend and retract through the parenchyma (Nimmerjahn et al., 2005), dynamically surveying the brain parenchyma. In addition, the assembly of neuronal circuits requires a delicate balance between synaptic formation and elimination. Microglia play a defined role in this process by eliminating excess synapses (Ferro et al., 2018). For instance, during brain development, microglia contribute to developmental synaptic remodeling *via* engulfing synapses from less active neurons (Wu et al., 2015), and thereby, promoting formation of precise neural circuitry (Li et al., 2020). In parallel, microglia dynamically investigate synapses in the adult brain in an activity-dependent manner and play a vital role in neuronal homeostasis and cognitive repair (Bartels et al., 2020). Dysphagocytosis of microglia may lead to synaptic over-pruning or inadequate pruning, which may be a direct culprit in neurodevelopmental and neuropsychiatric disorders such as autism and schizophrenia (Edmonson et al., 2016; Druart and Le Magueresse, 2019). Furthermore, microglia can also normalize dendritic spine increment in an experience-dependent manner (Tuan et al., 2021).

In pathological conditions such as infection, trauma, or cerebral ischemia, microglia rapidly transform from the resting state to the activated state, presenting a typical “amoeboid,” which is characterized by an enlarged cell body, shortened and thickened branches (Wolf et al., 2017), followed by migrating to the inflammatory site and secreting cytokines, chemokines, and neurotoxins, such as reactive oxygen species (ROS), thereby survey surrounding axons and invading pathogens.

Microglia dynamically switch between two polarized states, namely the M1 phenotype (pro-inflammatory) and the M2 phenotype (anti-inflammatory) and are involved in different CNS diseases such as cerebral ischemia, traumatic brain injury, and neurodegenerative diseases such as Alzheimer's disease (AD) and Parkinson's disease (Colonna and Butovsky, 2017; Li and Barres, 2018; Prinz et al., 2019). Microglia can recognize pathogens and other external stimuli on account of a rich array of receptors they express, including toll-like receptors (TLRs) (Li Y. et al., 2021), Fc receptors (Fuller et al., 2014), Ig-superfamily receptors such as triggering receptors expressed on myeloid cells 2 (TREM2) (Zhao et al., 2018; Andreone et al., 2020), scavenger receptors (Wilkinson and El Khoury, 2012), complement receptors (Werneburg et al., 2020), and fractalkine receptors (Gunner et al., 2019).

Compared to brain surgery and medication, exercise therapy is largely non-invasive and free from side effects. A large number of investigations have reported that exercise can induce structural and functional plasticity of the injured CNS, such as promoting the repair of injured neurons and the reconstruction of compensatory neural circuits (Mahalakshmi et al., 2020). This is especially one of the widely used neurorehabilitation methods after stroke (Billinger et al., 2014). Moreover, exercise can regulate brain function by acting on functional changes in microglia. Multiple animal studies have identified the regulation of exercise on the quantity, morphology, phenotype cytokine, and neurotrophic factor expression of microglia in several neurological disorders (Lu et al., 2017; Pignataro et al., 2021; Zhang et al., 2022). Physical exercise also mediates the crosstalk between microglia and astrocytes/neurons to enhance neuroplasticity. For example, the CX3CL1-CX3CL1R axis and the CD200-CD200R axis were more responsive to the same stimulus in the exercised animals than in the sedentary animals (Sung et al., 2012; Fleshner et al., 2014). The molecular mechanisms involved in the crosstalk between astrocytes/neurons and microglia have been elaborated on in recent reviews (Li F. et al., 2021). Mee-Inta et al. (2019) also provided a detailed review of the effect of exercise on microglia activation and its possible regulatory mechanisms. Moreover, Consorti et al. (2021) have reviewed the peripheral mechanisms that drive physical activity to influence the brain, in particular, the physiological processes that transform sensory input in muscles and joints into the activation of brain molecular pathways associated with neuroplasticity.

However, a few reports further summarize microglia functions, such as phagocytosis and environmental surveillance, and the effect of physical exercise on them. As an example, recent studies have shown that exercise reverses behavioral abnormalities in mouse models of autism spectrum disorder by stimulating microglia to engulf excess synapses (Andoh et al., 2019). Treadmill exercise preconditioning for 6 weeks promoted recovery of intracerebral hemorrhage mice with neurological deficits, reduced lesion size, and increased microglial phagocytosis (Kinoshita et al., 2021). This study discusses the possible mechanisms of microglia phagocytosis regulated by exercise. How signals generated by muscle movement are transmitted from the periphery to the center is not explained here, even though it is a crucial and complex process.

Here, we summarize recent knowledge on factors regulating microglial phagocytosis, including TREM2, lipid metabolism, metabolic reprogramming, complement, chemokine receptor, noradrenaline (NA), interleukin (IL)-33/ST2, and TAM system (Table 1). Pharmacological or exercise interventions through some of these factors may contribute to preventing neurodevelopmental dysfunction mediated by microglial phagocytosis of excess living neurons and synapses or neurodegeneration and neuropsychiatric disorders induced by microglia phagocytic dysfunction. Physical exercise has been shown to reverse microglial phagocytosis by modulating several factors. Through this review, we aim to make an overall overview of the molecular mechanism controlling microglia phagocytosis and provide new targets and ideas for physical exercise to promote neuroplasticity from the perspective of microglial phagocytosis.

**Table 1:** Regulation mechanism and exercise participation of microglia phagocytosis in different disease models.

	**Objects**	**Effects**	**Models**	**Disease model**	**Exercise participation**	**References**
TREM2	Synapses	Limits astrocyte uptake of synapses.	1-month-old mice	During development	N/A	Jay et al., 2019
	Neuronal debris	Mediates cell survival, phagocytosis, processing of neuronal debris, and lipid metabolism.	Human iMG	N/A	N/A	Andreone et al., 2020
	Aβ	Facilitates Aβ uptake by microglia and human macrophages through interaction with lipoproteins.	An unbiased protein microarray screen	AD	N/A	Yeh et al., 2016
	Myelin	Regulates cholesterol transport and metabolism at the transcriptional level.	Mice; iMG	Chronic demyelination model	N/A	Nugent et al., 2020
	N/A	16 weeks of physical exercise increases sTREM2 in CSF of AD patients.	198 patients with AD	AD	Treadmill, stationary bike, cross trainer	Jensen et al., 2019
	N/A	Three months of running wheels inhibits TREM2 shedding and maintains TREM2 protein levels.	APP/PS1 mice	AD	Running wheels	Zhang et al., 2022
Lipid metabolism	Aβ	Acetate, as the essential microbiome-derived SCFAs, modulates microglial phagocytosis of Aβ.	Germ-free WT mice; 5xFAD mice	AD	N/A	Erny et al., 2021
	Myelin	TREM2 regulates cholesterol transport and metabolism at the transcriptional level.	Mice; iMG	Chronic demyelination model	N/A	Nugent et al., 2020
Metabolic reprogramming	Aβ	The conversion of microglia to glycolysis leads to microglia phagocytic dysfunction and Aβaccumulation.	APP/PS1 mice	AD	N/A	McIntosh et al., 2019
	Aβ	Aβ induces metabolic reprogramming of microglia from OXPHOS to glycolysis.	5xFAD mice	AD	N/A	Baik et al., 2019
	Aβ	Metabolically inefficient glycolysis reduces the phagocytosis of microglia to Aβ.	Primary microglia in culture	N/A	N/A	Rubio-Araiz et al., 2018
	N/A	Glycolysis, glycolytic capacity and PFKFB3 are significantly increase in microglia from aged animals. Exercise ameliorates these effects and increases the phagocytic capacity of cells.	Aged (18 months old) C57BL/6 mice	N/A	Treadmill	Mela et al., 2020
Complement receptors						
C1q	Synapses	Mediates the elimination of redundant synapses in the retina during geniculate neuron development and in the early stage of glaucoma.	Mice with glaucoma	During development; glaucoma	N/A	Stevens et al., 2007
C3	Synapses	Viral overexpression of the complement inhibitor Crry at C3-bound synapses decreases microglial engulfment of synapses.	Postmortem human MS tissue, a preclinical non-human primate model of MS, and two rodent models of demyelinating disease	MS	N/A	Werneburg et al., 2020
C3	Presynaptic terminals	Viral infection of adult hippocampal neurons induces complement-mediated elimination of presynaptic terminals.	Murine and human West Nile virus (WNV) neuroinvasive disease post-mortem samples	Neuroinvasive infection	N/A	Vasek et al., 2016
C3	Synapses	Complement activation and opsonization of hippocampal synapses direct ongoing microglia-dependent phagocytosis of synapses.	Mice	Stroke	N/A	Alawieh et al., 2020
C1q and C3	Synapses	Mediate microglial phagocytosis of hippocampal synapses.	Postmortem of 31 MS donors	MS	N/A	Michailidou et al., 2015
Chemokine receptors						
CX3CR1	Synapses	Mediates whisker removal-induced synaptic elimination.	Mice	Sensory lesioning	N/A	Gunner et al., 2019
CCR4	Hematoma	CCL17 therapy promotes early hematoma regression after intracerebral hemorrhage through the CCR4/ERK/Nrf2/CD163 pathway.	Mice	ICH	N/A	Deng et al., 2020
CXCR3	N/A	Attenuates LPS-induced microglial phagocytosis and nitric oxide production in microglia and BV-2 cells.	Primary cortical neurons, astrocyte and microglia in culture; mice	Entorhinal cortex lesion model	N/A	de Jong et al., 2008
Adrenergic receptors	Aβ	NA in locus ceruleus projection areas facilitates microglial migration and phagocytosis in AD, thereby contributing to Aβ clearance.	APPV717I-transgenic mice; APP/PS-1-transgenic mice; Primary microglia in culture	AD	N/A	Heneka et al., 2010
	N/A	Both the activity of noradrenaline neurons in locus coeruleus and the expression of adrenergic receptors in healthy rats can by regulated by wheel running.	Rats	N/A	Wheel running	Van Hoomissen et al., 2004
	Synapses	VE significantly normalizes the SD-induced dendritic spine increment and maintains the microglial phagocytic ability.	Mice	Adolescent 72 h SD model	VE	Tuan et al., 2021
IL-33/ST2	ECM	Instructs microglial engulfment of ECM.	Mice	N/A	N/A	Nguyen et al., 2020
	N/A	IL-33-ST2-Akt signaling axis supports metabolic adaptation and phagocytosis of microglia.	Mice	During development	N/A	He et al., 2022
	Aβ	Ameliorates Aβ pathology by reprogramming microglial epigenetic and transcriptomic profiles to induce a microglial subpopulation with enhanced phagocytic activity.	APP/PS1 transgenic mice	AD	N/A	Lau et al., 2020
	Hematoma	IL-4/STAT6/ST2 signaling mediates microglia/macrophages phagocytosis of red blood cells.	Young and aged male and young female mice	ICH	N/A	Xu et al., 2020
TAM system	Aβ	TAM-driven microglial phagocytosis does not inhibit, but rather promotes, dense-core plaque development.	APP/PS1 mouse	AD	N/A	Huang et al., 2021
	Viral	Inhibition of phospholipid scramblase 1 (PLSCR1) activity prevents PtdSer externalization and enables months-long protection from microglial phagocytosis.	Mice	Viral infection	N/A	Tufail et al., 2017
	Synapses	Local externalization of PtdSer mediates developmental synaptic pruning by microglia.	Primary neurons and microglia in culture; mice	N/A	N/A	Scott-Hewitt et al., 2020
	Synapses	PtdSer is precisely exposed to the tip of the outer segment of the photoreceptor, which drives the RPE cells to engulf the local membrane segment.	Cell culture of porcine	N/A	N/A	Ruggiero et al., 2012
Negative regulators of phagocytosis						
CD22	Myelin debris; Aβ; α-synuclein fibrils	Mediates the anti-phagocytic effect of α2,6-linked sialic acid.	Aged (18–24 months old) and Young (2–4 months old) mice; primary microglia in culture	N/A	N/A	Pluvinage et al., 2019
CD47	Synapses	Protects synapses from excessive pruning during. development.	Primary neurons and microglia in culture; AD (APPswe and PSEN1dE9 in a single locus) mice	AD	N/A	Ding et al., 2021
CD47	Synapses	Prevents excess microglial phagocytosis.	Mice	N/A	N/A	Lehrman et al., 2018

*CSF, Cerebrospinal fluid; AD, Alzheimer's disease; MS, Multiple sclerosis; ICH, Intracerebral hemorrhage; Aβ, Amyloid beta; ECM, Extracellular matrix; SCFAs, Short-chain fatty acids; LPS, Lipopolysaccharide; NA, Noradrenaline; VE, Voluntary exercise; SD, Sleep deprivation; PtdSer, phosphatidylserine; RPE, Retinal pigment epithelial; iPSCs, Induced pluripotent stem cells; iMG, iPSCs-derived microglia cells*.

## TREM2

TREM2, an immunoglobulin superfamily receptor, was initially identified as a gene closely associated with the risk of AD in the Genome-wide association (GWAS) study (Jansen et al., 2019). TREM2 is expressed predominantly by microglia in the CNS (Colonna and Wang, 2016). TREM2 enhances microglia phagocytosis of Aβ plaques and ameliorates AD symptoms. During development, microglia rely on TREM2 to restrict the uptake of synapses by astrocytes while there are additional mechanisms that restrict glial cells from improperly eliminating synapses in adulthood. But a high-fat diet is sufficient to restart synaptic loss regulation dependent on TREM2. Defects in TREM2 affect the number of synapses and glial phagocytosis of synaptic buttons in multiple brain regions (Jay et al., 2019). Mechanically, TREM2 binds to its associated immune ligands and signals through tyrosine-based activation motifs (ITAM), such as the adapter protein DNAX activation proteins 12 (DAP12) that recruits downstream tyrosine kinase SYK, ultimately promoting chemotaxis, phagocytosis, and proliferation of microglia (Ulland and Colonna, 2018). The TREM2-DAP12 interaction also activates PI3K/AKT, followed by blocking the MAPK cascade. These signaling pathways ultimately inhibit TLR4-driven inflammatory responses in microglia (Mecca et al., 2018). In good correlation to these findings, Andreone et al. recently confirmed that PLCγ2 regulates microglial survival, myelin phagocytosis, neuronal debris processing, and lipid metabolism as a downstream molecule in TREM2. PLCγ2 also signals TLRs independently of TREM2, mediating an inflammatory response (Andreone et al., 2020). TREM2 has been reported to drive microglia to gather around amyloid-β (Aβ) plaques and engulf them (Yeh et al., 2016). Deletions or mutations in TREM2 exacerbate Aβ toxicity and increase the risk of AD. In the AD model, mice with TREM2 R47H mutations had reduced microglia proliferation, activation, and recruitment to Aβ plaques (Hall-Roberts et al., 2020).

In addition, TREM2 also indirectly affects phagocytic function by regulating microglia metabolism. It has been reported that TREM2 senses lipids and mediates myelin phagocytosis. TREM2 has been identified as a key transcriptional regulator of lipid transport and metabolism. Ligands of TREM2 include lipoprotein particles, such as LDL as well as apolipoproteins (Yeh et al., 2016). Both Apolipoprotein E (APOE) and Aβ oligomers are able to bind to and activate TREM2 (Gratuze et al., 2018; Zhao et al., 2018). Yeh et al. (2016) found that both TREM2 deficiency and APOE deficiency inhibited plaque-associated microglia proliferation in a mouse model of AD. Recent studies have also reported that microglia with TREM2 mutations or lack of TREM2 can engulf myelin fragments but fail to clear cholesteryl ester due to impaired cholesterol transport while accumulation of cholesteryl ester was also observed in APOE-deficient glial cells (Nugent et al., 2020). Overall, TREM2 plays a key role in microglia recruitment, phagocytosis of Aβ plaques, lipid transport, and metabolism. All of these studies suggest that increasing TREM2 expression is an effective treatment to delay the onset of AD (Zhong et al., 2017) and demyelinating disease. Intriguingly, interactions among microglia, neurons, and oligodendrocytes contribute to the selective phagocytosis of myelin sheaths, that is, neuronal activity competitively attracts microglia to the neuronal cell body, thus occupying the microglia used for myelin phagocytosis, while reduced neuronal activity promotes myelin phagocytosis (Hughes and Appel, 2020). Whether this neuronal activity-dependent myelin remodeling cooperates with TREM2 needs to be further verified.

Levels of soluble TREM2 in the cerebrospinal fluid (CSF) were significantly increased in AD patients with long-term physical exercise (Jensen et al., 2019). Similarly, a recent study reported that long-term voluntary running exercise ameliorated cognitive impairment in APP/PS1 mice by inhibiting TREM2 abscission and maintaining TREM2 protein levels while promoting microglial glucose metabolism and hippocampal morphological plasticity in AD mice (Zhang et al., 2022). In brief, exercise regulates microglial phagocytosis by targeting TREM2 directly or indirectly.

## Lipid metabolism

In mice and human brains, lipid droplets accumulate significantly in microglia with age. These cells produce an abundance of ROS (Yousef et al., 2019; Marschallinger et al., 2020), secrete pro-inflammatory cytokines, and are deficient in phagocytosis, known as lipid droplet-accumulating microglia (LDAM). More than half of the microglia were found in the LDAM state in the aged hippocampus (Marschallinger et al., 2020).

In activated microglia, metabolic pathways are significantly altered. Microglia activation, phagocytosis, migration, and release of inflammatory factors may be controlled by microglia lipid metabolism (Chausse et al., 2021). *In vitro* studies indicated that inhibition of lipid droplet formation significantly increased the phagocytosis of BV2 cells, implying that lipid droplets had an adverse effect on phagocytosis. Intriguingly, LDAM contain a large number of lysosomes, which accumulate near the lipid droplets. Therefore, it is hypothesized that in LDAM, lysosomes are more inclined to degrade lipid droplets than the substances being swallowed, resulting in impaired phagocytosis. In addition, there is evidence of lysosome dysfunction in senescent microglia (Mosher and Wyss-Coray, 2014). Therefore, in elderly LDAM, lysosome dysfunction may lead to both phagocytosis impairment and lipid droplet accumulation, which further damages the phagocytosis.

Consistent with this, phagocytosis in macrophages depends on accessible free fatty acids, which are released when the lipid droplets degrade (Chandak et al., 2010). The phagocytic activity of lipid droplet-enriched foam macrophages was lower than that of lipid droplet-free macrophages in atherosclerotic lesions (Chinetti-Gbaguidi et al., 2011). In parallel, recent studies have suggested that acetate, a critical microbiome-derived short-chain fatty acid, regulates disease progression in the mouse model of AD by inhibiting microglial phagocytosis of amyloid-beta (Erny et al., 2021). This suggests that the normal phagocytosis of microglia requires the assistance of increased lipid metabolism (Loving and Bruce, 2020).

At the transcriptional level, genes such as *APOE, TREM2*, and Lipoprotein Lipase (*LPL* are increased in microglia both during development and in disease states (Loving and Bruce, 2020). Regulation of lipid metabolism in TREM2 has been described above. In parallel, Nugent et al. reported that TREM2 upregulates *APOE* and other microglial genes associated with chronic demyelinating injury (Nugent et al., 2020). On the whole, there are some interactions among various lipid metabolism-related genes in microglia, which jointly regulate phagocytosis in different pathological conditions. The specific mechanism by which lipid droplets affect phagocytosis remains to be studied. However, whether exercise training may affect microglial phagocytosis by regulating lipid metabolism remains unclear.

## Metabolic reprogramming

Metabolic reprogramming is a process in which cells exhibit different metabolic characteristics in response to changes in the microenvironment in order to provide energy and biological matter (Mo et al., 2019). Immune cells have a variety of functions, and they would adjust their alternative metabolic pathways in real-time to meet their energy needs based on cell phenotypes. There is increasing evidence that metabolic reprogramming is a key energy basis for the immune response of microglia. Microglia homeostasis depends on oxidative metabolism, while the metabolism of microglia shifts to glycolysis under pro-inflammatory stimulation (Lynch, 2020). The transition from oxidative phosphorylation (OXPHOS) to glycolysis interferes with its phagocytosis. An *in vitro* study showed that the combination of interferon -γ (IFN-γ) and Aβ can induce glycolysis in microglia and its ability for phagocytosis and chemotaxis is decreased (McIntosh et al., 2019). Besides, TREM2, as mentioned above, is thought to play a key role in regulating microglial metabolic patterns.

Although glycolysis is not as efficient at producing ATP as OXPHOS, glycolysis has a glucose metabolism rate 10–100 times faster than OXPHOS, which enables microglia to complete energy-intensive processes such as phagocytosis. However, only 2 molecules of ATP are produced per glucose due to inefficient glycolysis metabolism, while oxidative metabolism produces about 36 molecules of ATP, so cellular fatigue may lead to the occurrence of functional deficits. Baik et al. have reported acute administration of Aβ-activated microglia, along with metabolic processes from OXPHOS to glycolysis. This process relies on the mTOR-HIF-1a pathway. However, the physiological processes of microglia such as cytokine secretion and phagocytosis are defective in the long run. IFN-γ administration, as a potential treatment, can restore microglia glycolytic metabolism and immune function (Baik et al., 2019). In short, regulating microglia metabolism may be a potential therapeutic strategy.

In contrast, an *in vitro* study showed that anti-TLR2 antibodies increased the phagocytosis of Aβ by LPS-stimulated microglia, which is associated with inhibition of the activity of the key glycolysis enzyme fructose-6-phospho-2-kinase/fructose-2, 6-bisphosphatase (PFKFB3), and the metabolic pattern of microglia shifted from glycolysis to oxidative metabolism (Rubio-Araiz et al., 2018). The glycolysis, glycolytic capacity, and PFKFB3 were significantly increased in elderly microglia, and exercise could alleviate the above effects and increase the phagocytosis ability (Mela et al., 2020). In brief, exercise reduces the dependence of microglia on metabolically inefficient glycolysis.

## Complement receptors

C1q and C3, the components of the classical complement cascade, bind to the surface of invading pathogens, which are then cleared by phagocytes expressing corresponding complement receptors in the peripheral immune system (Morgan and Kavanagh, 2018; Reis et al., 2019). Similarly, in the CNS, microglia rely on classical complement cascades to mediate phagocytic signaling, removing excess synapses. Mice with C3, C1q, or complement receptor 3 (CR3) deficiency had persistent synaptic pruning defects, and about 50% of microglial synaptic phagocytosis was blocked in CR3-deficient mice (Schafer et al., 2012; Hong et al., 2016). In parallel, C1q and C3 are also localized in the visual thalamus of developing rodents (Stevens et al., 2007). Microglia expressing CR3 engulf these complement-associated synapses. On the contrary, viral overexpression of the complement inhibitor Crry protects visual function by decreasing microglial engulfment of synapses (Werneburg et al., 2020).

Similar molecular mechanisms also modulate early synaptic loss in neurodegenerative model mice. For example, in demyelinating disease, microglia eliminate synapses through selective complement cascades (Vasek et al., 2016). In a mice model of AD, aggregation of Aβ can activate complement, which influences microglial phagocytosis through complex crosstalk with TLR and inflammasome signaling pathways, thereby, regulating synaptic pruning and loss (Yang et al., 2020). After both reperfused and non-reperfused stroke, complement activation directs ongoing microglia-dependent phagocytosis of synapses for at least 30 d after stroke, leading to a loss of synaptic density that is associated with cognitive decline (Alawieh et al., 2020). Another postmortem report of a multiple sclerosis (MS) patient also showed that C1q and C3 co-located with synaptic proteins in the hippocampus (Michailidou et al., 2015).

The specific mechanism that transforms the combination of complement and complement receptor into the phagocytosis of the synaptic remains unclear. Some studies have suggested that this is associated with Ca^2+^ influx into boutons in activated neurons, followed by the activation of local Ca^2+^-dependent scramblases, which lead to the externalization of phosphatidylserine (PtdSer). The externalized PtdSer eventually binds to C1q to trigger synaptic pruning (Païdassi et al., 2008; Lemke, 2017). It is not clear whether the binding of C3 to its receptor requires PtdSer, but Allendorf et al. have suggested that increased sialidase activity in activated microglia leads to desialylation in the cell surface, which stimulates microglia to phagocytose neurons in a CR3-dependent manner (Allendorf et al., 2020).

It has been reported that chronic aerobic exercise can maintain the integrity and function of neurovascular units in aging mice by reducing C1q^+^ microglia and increasing neuroplasticity (Soto et al., 2015). Furthermore, physical exercise may promote cellular crosstalk between microglia and neurons through complement molecules (Li F. et al., 2021).

Most of these studies have been conducted in the mouse model. However, it has been suggested that mouse models cannot fully solve this problem because human microglia are markedly different from rodent microglia, with higher complement components and multiple important gene expressions during brain development (Gosselin et al., 2017). There is no doubt that further research is needed to clarify this point.

## Chemokine receptors

CX3CL1 is one of the critical chemokines and is mainly expressed in neurons in a soluble or membrane-bound state. CX3CR1, a G protein-coupled chemokine receptor, which is highly expressed in microglia (Wolf et al., 2013), keeps microglia homeostasis by binding to CX3CL1 (Madrigal et al., 2017). The CX3CR1/CX3CL1 axis plays an important role in maintaining CNS homeostasis under physiological conditions.

In the developing thalamic cortex, microglia accumulate at synaptic concentration sites and control synaptic maturation through the CX3CR1/CX3CL1 signaling pathway. CX3CR1-deficient mice showed delayed functional maturation of postsynaptic glutamate receptors (Paolicelli et al., 2011; Hoshiko et al., 2012). In addition, artificial removal of whisker induces microglial synaptic phagocytosis and synaptic elimination during development through the CX3CR1-CX3CL1 rather than the CR3 signaling pathway. Gunner et al. showed that *Adam10*, a gene that encodes a metalloproteinase, plays an important role in cleaving CX3CL1 into a secretory phenotype, and the cleaved CX3CL1 then binds to CX3CR1 and initiates phagocytosis and remodeling of synapses by microglia (Gunner et al., 2019).

Tau and CX3CL1 interact competitively with CX3CR1, and the expression level of CX3CL1 is reduced in the AD brain, so CX3CR1 directly binds to Tau and promotes its internalization and uptake (Chidambaram et al., 2020). In intracerebral hemorrhage model mice, microglial phagocytosis is an important channel to promote hematoma regression. It has been reported that treatment with CCL17, a specific ligand of CCR4, promotes hematoma resolution through the CCR4/ERK/Nrf2/CD163 pathway, thereby, improving neurological function, which is associated with microglia-mediated erythrocyte phagocytosis and clearance of tissue debris (Deng et al., 2020). Additionally, CXCR3b, another splicing variant of CXCR3 expressed in human microvascular endothelial cells, has been shown to bind to CXCL4, which is mainly derived from microglia under neurodegenerative conditions *in vitro* and *in vivo*. CXCL4 reduces LPS-induced phagocytosis of microglia and BV-2 cells and the production of nitric oxide (de Jong et al., 2008). Overall, microglia in various disease models appear to activate different chemokine receptors to demonstrate phagocytosis.

Physical exercise is reported to ameliorate cognitive function and neuroplasticity in depressed mice through microglia-mediated CX3CL1-CX3CR1 signaling (Eyre et al., 2013). Similarly, it has been suggested that physical exercise could accelerate the response of the CX3CL1-CX3CR1 axis to stressors and rapidly quieten the activated microglia, thus avoiding the negative cognition-related effects of stress (Fleshner et al., 2014). However, whether exercise training may affect microglial phagocytosis by regulating CX3CL1-CX3CR1 needs further study.

## Adrenergic receptors

NA in the cortex was derived only from noradrenergic neurons in the locus coeruleus (Berridge and Waterhouse, 2003). Although adrenergic receptors are widely expressed in the neurons and glia (O'Donnell et al., 2012), microglia are the major sites of NA signaling in the cortex. Within the cortex, there is considerable evidence that β1-adrenergic receptor (β1-AR) and β2-adrenergic receptor (β2-AR) are the only functionally significant adrenergic receptors in microglia (Heneka et al., 2010). Moreover, in the non-injured brain, microglia are highly enriched with β2-AR relative to other CNS cell types (Zhang et al., 2014). NA acts as a powerful regulator of microglia function in physiological or pathological settings, including microglial processes motility, arborization, and contact with dendrites (Liu et al., 2019; Stowell et al., 2019). Previous studies have also shown that microglia β2-AR are essential for microglia locomotion, migration, phagocytosis, proliferation, and inflammatory responses (O'Donnell et al., 2012).

In models of AD, there is evidence that the non-selective β-adrenergic receptor agonist isoproterenol indirectly enhances the phagocytosis of Aβ plaques (Heneka et al., 2010) in addition to promoting microglia migration toward Aβ plaques in culture (Kettenmann et al., 2011). Degeneration of the locus coeruleus may contribute to the pathogenesis of AD (Kalinin et al., 2007).

Both the activity of norepinephrine neurons in locus coeruleus and the expression of adrenergic receptors in healthy rats can be regulated by wheel running (Van Hoomissen et al., 2004). Furthermore, voluntary exercise is beneficial to the normalization of dendritic spine's increment and the maintenance of microglial phagocytosis in sleep-deprived mice. The modification of norepinephrine signaling in the CNS may be responsible for the preventive effect of voluntary exercise on microglia and neuron dysfunction induced by sleep deprivation (Tuan et al., 2021). Yet, the impact of physical exercise on the norepinephrine system during brain development remains elusive.

## IL-33/ST2

IL-33 is normally inactive in the nucleus and acts as a transcriptional regulator. When cells are subjected to mechanical stress, inflammatory cytokines stimulation, or necrosis, IL-33 is released into the extracellular space and hydrolyzed. Subsequently, the hydrolyzed IL-33 signals adjacent immune cells expressing ST2 receptors in an autocrine/paracrine manner (Altara et al., 2018). During the early stages of synaptic maturation after birth, IL-33 expression is increased in astrocytes in the gray matter of the spinal cord and thalamus, where most synapses are concentrated. In this process, IL-33/ST2 is primarily responsible for driving microglial synapse engulfment and restricted excitatory synapse numbers (Sun et al., 2021). On the other hand, IL-33 is widely expressed in adult brain regions such as the corpus callosum, hippocampus, thalamus, and the granular layer and white matter of the cerebellum. It is also primarily located in astrocytes in the mouse brain and spinal cord (Pichery et al., 2012).

Interestingly, a recent study has shown that most IL-33-expressing cells in the adult hippocampus are neurons and are primarily responsible for instructing microglial engulfment of the extracellular matrix (ECM) and reshaping synapses, which are required for memory consolidation. IL-33 deficiency leads to impaired phagocytosis of ECM by microglia, along with the accumulation of ECM proteins, especially around the synapses and dendritic spines. Treatment with exogenous ECM enzymes contributes to restoring the number of dendritic spines in IL-33-deficient mice (Nguyen et al., 2020). Neuronal IL-33 expression levels are progressively decreased with age, which is consistent with deficits in memory accuracy and the accumulation of ECM around synapses (Végh et al., 2014b). Notably, elimination of ECM or administration of exogenous recombinant IL-33 reverses cognitive decline and memory deficits in the mouse model of AD (Végh et al., 2014a; Fu et al., 2016). Besides, He et al. demonstrated the functional combination of mitochondrial bioenergetics and the phagocytic activity of microglia. They propose a microglia-astrocyte signaling pathway, the IL-33-ST2-Akt signaling axis, which is a crucial pathway for maintaining metabolic adaptation and phagocytic function of microglia during early development and is associated with neurodevelopment and neuropsychiatric disorders. Mechanistically, the IL-33-ST2 axis promoted microglia phagocytosis and energetic metabolism in an AKT-dependent manner. PTEN or SHIP-1 negatively regulates microglial phagocytosis and energy metabolism by inhibiting AKT activation. Inhibition of mitochondria-dependent energy metabolism reduces the ability of microglia to phagocytic synaptosomes (He et al., 2022).

IL-33 and ST2 can also act on their downstream molecules alone and indirectly affect microglial phagocytosis. For example, studies have found that injection of IL-33 in AD transgenic mice enhanced Aβ clearance by reprogramming microglia epigenetics and transcriptome profiling, thereby alleviating AD pathology (Lau et al., 2020). Similarly, in mouse models, microglia promote hematoma regression and functional recovery after intracerebral hemorrhage through the IL-4/STAT6/ST2 signaling pathway. Both *in vivo* and *in vitro* experiments showed that STAT6-KO impaired the ability of microglia to phagocytose red blood cells (Xu et al., 2020).

In general, IL-33/ST2 is closely related to microglial energy metabolism, phagocytosis, epigenetics, and transcriptome profiles. A further understanding of how these mechanisms translate between physiological states and different pathologies requires a better understanding of the temporal process of IL-33 expression, defining the dynamics of IL-33 expression at a more precise time and individual neuron level, and mastering the regularity of IL-33 protein release, which are important areas for future research. So far, no studies have examined the impact of exercise training on ST2 in microglia.

## TAM system

Studies have shown that the TAM system is an important mediator for microglia to recognize and phagocytose apoptotic cells (ACs) (Lu and Lemke, 2001) and amyloid plaques (Huang et al., 2021). TAM proteins include cell surface receptor tyrosine kinases (RTKs)- Tyro3, Axl, and Mer (encoded by Mertk) (Lemke, 2013). Human and mouse microglia normally express high levels of Mer and low levels of Axl (Fourgeaud et al., 2016; The ImmGen Consortium, 2016).

PtdSer is present in many different membranes in every cell of the body, including the endoplasmic reticulum, mitochondria, Golgi apparatus, and plasma membrane. In the plasma membrane of normal cells, PtdSer is almost entirely confined to the inner leaflet of the lipid bilayer (Leventis and Grinstein, 2010; van Meer, 2011). When programmed death is initiated, or in some pathological cases, local externalization of eat-me signals such as PtdSer in specific areas of the cell surface allows microglia to recognize and phagocytize pruning of these “locally dead” domains or cells with metabolic damage that are still alive (Lemke, 2019). Inhibition of phospholipid scramblase 1 (PLSCR1) activity avoided intracellular calcium imbalance, prevented extravasation of PtdSer, and protected target cells from microglial phagocytosis for several months (Tufail et al., 2017).

However, PtdSer activates TAM receptors through bridging molecules such as growth arrest-specific (GAS6) and protein S (PROS1) rather than binding directly to TAM proteins (Dransfield et al., 2015). Therefore, GAS6 and PROS1 may be regarded as co-receptors. Specifically, the carboxy-terminal SHBG domain of these ligands binds to Axl and Mer on microglia, whereas the gamma- carboxyglutamic acid (GLA) domain binds to PtdSer (Lemke, 2017).

During synaptic development, microglia engulf PtdSer-labeled hippocampal and retinogeniculate synapses. Blockade of the PtdSer-exposed pathway with Annexin V partially prevents synaptic elimination (Scott-Hewitt et al., 2020). In the APP/PS1 mouse model of AD, gene ablation of Axl and Mer causes microglia to fail to detect, respond to, organize, or engulf amyloid -β plaques properly (Huang et al., 2021). Furthermore, PtdSer and GAS6 were detected on all Aβ plaques surfaces (Huang et al., 2021). After Axl is activated, metalloproteinases cleft the extracellular domain of Axl exposed on the cell surface (Zagórska et al., 2014). Increased levels of the soluble outfield (sAxl) in CSF, combined with GAS6 complex, could predict disease progression and progression in human AD (Mattsson et al., 2013; Sainaghi et al., 2017). Interestingly, the Aβ that was swallowed by microglia mediated by TAM was not eliminated but deposited in the cells. These Aβ fibrils entering the lysosome are compacted into indigestible dense nuclear plaques (Huang et al., 2021). Similarly, the elimination of apoptotic neurons requires Mer and CR3, while Axl is negligible in this process (Anderson et al., 2022). During ACs phagocytosis, GAS6 and/or PROS1 act as a bridge between the externalized PtdSer on ACs and TAM receptors on microglia (Lemke, 2019).

It is worth noting that microglia can selectively phagocytose parts of the cell, instead of the whole cell. For instance, PtdSer is precisely exposed to the tip of the outer segment of the photoreceptor, which drives the retinal pigment epithelial (RPE) cells to engulf the local membrane segment (Ruggiero et al., 2012), while how PtdSer externalization is limited to the small membrane domain of cells remains unknown. It has been suggested that this is related to the local aggregation of Ca^2+^ channels in the plasma membrane, which locally activates Ca^2+^-dependent scramblases, thereby promoting the local cleavage of caspase 3 and Caspase 7 (Lemke, 2019). Further research is needed to provide evidence of those processes. Whether physical exercise would drive the externalization of PtdSer or the change of TAM protein expression needs further study.

## Negative regulators of phagocytosis

“Don't-eat-me” is a signal that inhibits the phagocytosis of immune cells. By combining the CRISPR–Cas9 knockout screens with RNA sequencing analysis, Pluvinage et al. screened out CD22, a typical B cell receptor, as a “Don't-eat-me” signal protein, which was upregulated in aging microglia. The anti-phagocytosis of α2, 6-linked sialic acid depends on CD22, which may cooperate with clearance pathways including the perivascular glymphatic (Kress et al., 2014) and meningeal lymphatic systems (Da Mesquita et al., 2018) to control the accumulation of debris and local cytokine concentrations in the aging brain. Inhibition of CD22 by antibody blocking or gene knockout promotes clearance of myelin fragments, Aβ, and α -synuclein fibrils by microglia *in vivo* (Pluvinage et al., 2019). CD47, a transmembrane protein, is another negative regulator of microglia phagocytosis. It is expressed in most mammalian cells, including neurons, and inhibits phagocytosis in neurodegenerative diseases through signal-regulatory protein alpha (SIRP α) on microglia (Ding et al., 2021). CD47 is also expressed at developing synapses and inhibits microglia-mediated synaptic clearance (Lehrman et al., 2018).

## Conclusion

**Figure 1:**
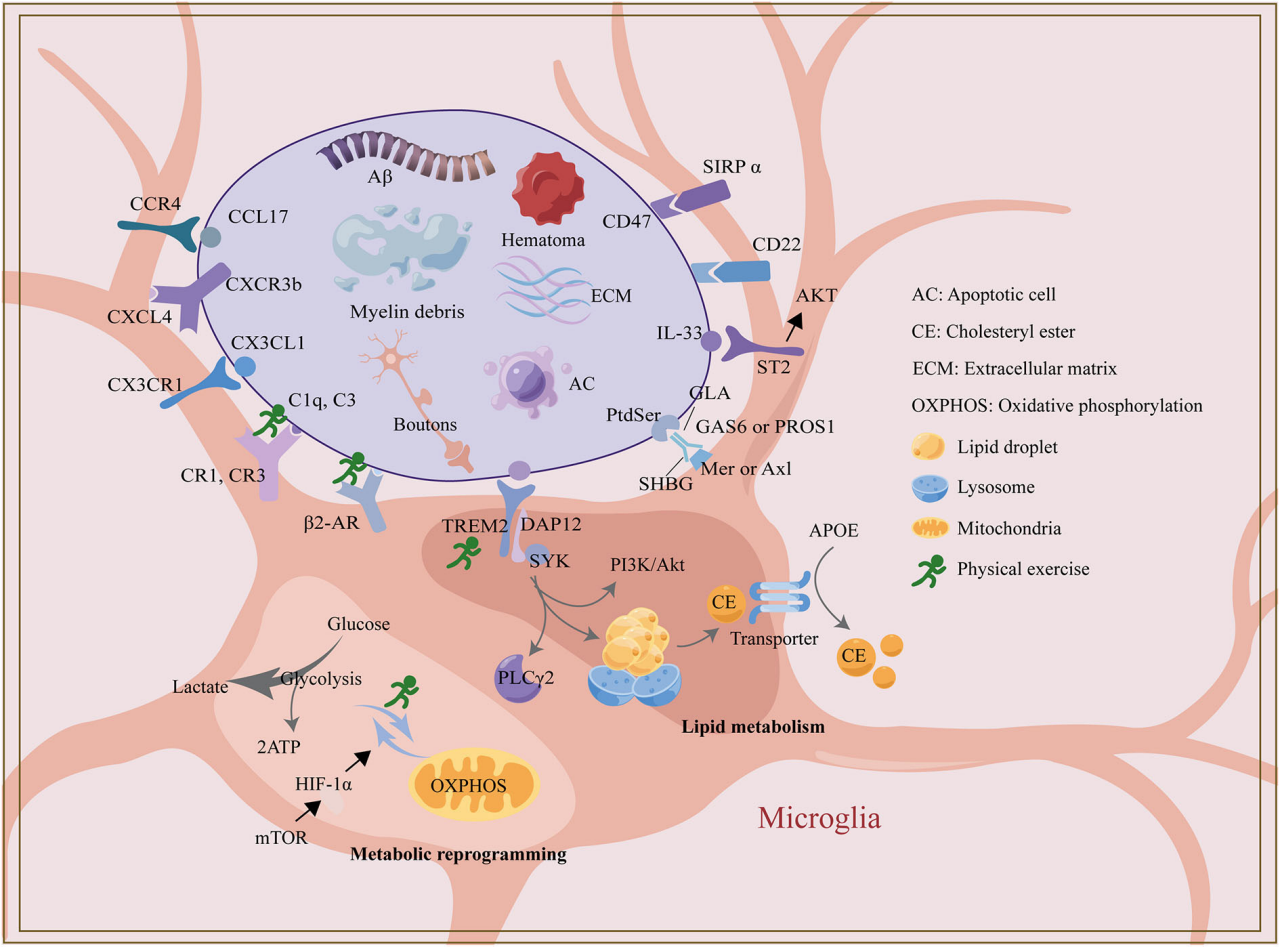
The regulatory mechanism of microglia phagocytosis and exercise.

Together, microglial phagocytosis is particularly important in developing synaptic pruning and amyloid clearance in AD mice while evidence for microglial phagocytosis in other disease models is lacking. Many scholars have conducted extensive research on the mechanism of microglial metabolic reprogramming, lipid metabolism, ligand, and receptor binding related to phagocytosis, which provides new targets and new ideas for the treatment of synaptic pruning deficiency and Aβ clearance disorder and also is one of the ways for some drugs and physical exercise to exert neural remodeling effect. TREM2 not only plays a role through its downstream tyrosine kinase SYK, but also indirectly regulates microglia lipid metabolism and glucose metabolism, which may be a vital intervention target for drugs or physical therapy. Based on the previous research, exercise training has been shown to influence microglial phagocytosis by modulating the glycolysis process and expression of TREM2, complement and adrenergic receptors. However, the specific cellular and molecular mechanisms of the effect of exercise training on microglia phagocytosis are still lacking solid evidence and in-depth discussion [Figure 1, created with Adobe Illustrator CC 2018 (USA) and Figdraw].

In summary, the phagocytosis levels of different microglia may be heterogeneous due to the expression levels of immune receptors on the cell surface and their metabolic status. Further research is needed to determine how microglia respond to the regulation of different immune pathways. Deciphering microglia heterogeneity may depend on a combination of single-cell sequencing techniques and targeted mouse models in further study. Furthermore, although microglia are the primary phagocytes scavenging cell debris in the CNS, a growing number of studies have reported astrocyte phagocytosis in microglia-deficient or dysfunctional mice. It has been suggested that astrocytes are on standby when microglia are damaged (Konishi et al., 2020). This compensatory mechanism may be conducive to maintaining or prolonging a healthy CNS, but the extent and duration of this compensation are unclear. However, what kind of “eat-me” signal drives astrocyte phagocytosis remains unclear, which may be a direction for further research.

## Author contributions

CL and YB had the initial idea for this article. CL and YW prepared figures and drafted the manuscript. YX and JHan performed the literature search. YB approved the final version of the manuscript and provided the funds. All authors critically revised the manuscript. All authors contributed to the article and approved the submitted version.

## Funding

This work was supported by the National Natural Science Foundation of China (grant number 82072540).

## Conflict of interest

The authors declare that the research was conducted in the absence of any commercial or financial relationships that could be construed as a potential conflict of interest.

## Publisher's note

All claims expressed in this article are solely those of the authors and do not necessarily represent those of their affiliated organizations, or those of the publisher, the editors and the reviewers. Any product that may be evaluated in this article, or claim that may be made by its manufacturer, is not guaranteed or endorsed by the publisher.
